# Development and validation of a job aid: Tool to reduce infections in home-based stroke

**DOI:** 10.4102/phcfm.v16i1.4221

**Published:** 2024-05-06

**Authors:** Violet K. Chikanya, Sindiwe James

**Affiliations:** 1 Department of Nursing Science, Faculty of Health Science, Nelson Mandela University, Port Elizabeth, South Africa

**Keywords:** infections, interventions, job aid, primary caregivers, stroke

## Abstract

**Background:**

Stroke patients who are discharged from hospital because of limited access to rehabilitation facilities are cared for by lay caregivers who at times have limited knowledge of infection prevention and control (IPC). User-friendly educational interventions can help bridge this knowledge gap and enhance safe care of these persons.

**Aim:**

To describe the development and validation of educational interventions for home-based stroke patients. The validation process enhanced the reliability and validity of the job aid resulting in standardised quality patient care of stroke patients.

**Setting:**

Mutasa district, Manicaland province, Zimbabwe.

**Methods:**

The systematic six steps in quality intervention development guided the development of the job aid. Graphic designers assisted with development of diagrams and annotations. A purposively selected eight-member panel of IPC expert reviewers was invited to validate the job aid using a standardised validation tool.

**Results:**

The panel agreed that the job aid’s title, target group and media of instruction were adequately explained, and the background could be easily understood during practice. The content was approved with some modifications on the description of instructions to caregivers. Seven reviewers agreed that the materials used ensured understandability, acceptability, practicability and usability of the educational interventions by caregivers, and one reviewer was neutral in commenting effectiveness of the job aid.

**Conclusion:**

The developed job aid addressed knowledge barriers in IPC for caregivers, and the reviewers confirmed that the developed job aid was adequate for effective use by lay home-based caregivers.

**Contribution:**

Utilisation of this intervention standardises patient care practices.

## Introduction

Globally, over 12.2 million people experience a stroke every year and over 101 million people are survivors.^1^ According to the World Stroke Organization’s (WSO) Global Stroke Fact Sheet 2022, the most updated stroke information, from 1990 to 2019, globally, the burden of stroke significantly increased with 70% increase in new strokes, 43% deaths from stroke, 102% prevalent strokes and 143% disability-adjusted life years (DALYS). Majority of the stroke burden (86% of deaths and 89% of DALYs) occur in low- and middle-income countries.^2^ The burden of stroke in Africa is increasing because of population growth and ageing.^3^

In Zimbabwe, stroke remains high and is among the top 10 causes of death. According to the latest World Health Organization (WHO) data published in 2020, stroke deaths have increased from 3.9% of the total deaths with an age-adjusted death rate of 63.38 per 100 000 in 2019 to 4.73% of the total deaths with an age-adjusted death rate of 93.42 per 100 000 in 2020.^4^

Infection in stroke patients can cause several health complications that become a challenge to families and communities that are staying and looking after these patients. Common health complications are but not limited to fever, hypoxia and electrolyte imbalance and may impair neural survival within the ischaemic penumbra,^5^ high incidences of pneumonia (30%) and urinary tract infections (UTIs) (30%).^6,7,8^ Most fearful of these infections after a stroke is an estimated 20% of mortality and considerable morbidity in stroke patients,^6,9^ and thus it is advisable to manage the condition effectively.

Although stroke is on the increase in Zimbabwe, it was noted that the incidence rate of stroke for the Mutasa district decreased from 7.31/100 000 to 2.01/100 000 between 2019 and 2022. However, a significant number of the new stroke patients developed chest infections (50.7%), skin infections (53.4%) and UTI (54.8%) after discharge.^10^ These infections are attributed to receiving non-proper care from caregivers who are not prepared to assist stroke patients with daily routines and mobility. The primary caregivers (PCGs) of stroke patients are not trained on stroke care and lack mentorship, which compromises the quality of care received by the stroke patients.^8,11^ The escalating number of complications in stroke cases calls for the establishment of stroke rehabilitation services such as stroke units. However, these services as well as stroke rehabilitation professionals are scarce in Africa.^12^ The lack of stroke rehabilitation facilities results in the family and informal caregivers bearing the responsibility of stroke patient post-discharge. Overall, PCGs lack knowledge on patient care leading to suboptimal services^13,14^ such as poor infection prevention and control (IPC) measures.

Furthermore, there are no specific educational interventions on IPC for PCGs caring for home-based stroke (HBS) patients, to ensure standardisation in training in IPC in the country. This results in an information gap, which leads to poorly executed stroke patient care and poor quality of life for the stroke patients.

In Zimbabwe after a brief hospital admission, stroke patients are often discharged home where they are nursed by caregivers who may be unprepared informal caregivers such as a family member or friend. Some of the caregivers are assuming the caregiver role for the first time and might lack the needed knowledge and skill for the role. Results from the empirical findings of a cross-sectional study conducted in Mutasa, Zimbabwe, indicated that there is a dearth of knowledge, ineffective practices of PCGs as well as limited information given by village health workers (VHWs) regarding prevention and control of infections in HBS patients.^15^ The results further indicated a need of educational interventions such as job aids that can be used to fill the knowledge gap.^15^ In response to this result, the authors recommended that the quality of care of HBS patients would be enhanced using job aids, which are visual support materials that provide the right kind of information using graphics and words in a simple and yet effective manner.^16^ The job aids allow the user to quickly access information required to perform a task with fewer errors and in less time.^17^

Job aids have been utilised in a variety of clinical and community settings; however, there is scarcity of literature showing the use of job aids in the management of stroke patients. Evidence has demonstrated that such job aids have been utilised in case management communication in children in HIV care settings in Uganda^18^ and HIV counselling for recipients of care on antiretroviral therapy (ART) in Zimbabwe.^19^ In addition, job aids have also been effectively used in antenatal care counselling.^16^ The Family Health International^20^ in its project utilised the community health worker Job Aids Booklet to enhance the support provided to community health workers (CHWs) as they offered family planning services.

This article aims to report on the development and validation of educational job aids for preventing and controlling infections among HBS patients, which was a result of an empirical study conducted by the authors of the current article.

It is anticipated that utilisation of the job aid will enhance the quality of home-based care services in particular for stroke patients in resource-limited settings. The lessons learnt from the use of this job aid will improve efficiencies in home-based care service delivery and trigger innovative ideas from researchers and community health practitioners. Utilisation of the job aid will promote standardisation in provision of care to HBS patients.

## Methods

### Study design and methods

The study was conducted in three phases. Phase One involved exploring and describing the knowledge and practices of PCGs on the prevention and control of infections among HBS patients.^15^ Data were collected from the PCGs and VHWs between 11 November and 10 December 2019. The research population in this study comprised VHWs and PCGs caring for stroke patients at home in the rural Mutasa district of Zimbabwe. The study participants were 200 PCGs of stroke patients and 200 VHWs who supervised the selected PCGs. Data were collected from the selected PCGs and VHWs using interviewer-administered and self-administered questionnaires, respectively. These data sets (from the two groups of participants) were captured by using Microsoft excel two templates and were analysed using a Visual Basic for Applications (VBA) package.

The study findings indicated that a significant number of PCGs had low knowledge levels on stroke and infections that frequently occur in stroke patients. About a third of PCGs did not know standard IPC precautions to prevent infections. A substantial proportion of the PCGs did not practice the recommended measures to prevent and control infections in HBS patients. Village Health Workers provided limited information on stroke and its complications of chest and skin infections and UTIs to PCGs. Only half of the VHWs disseminated information on IPC measures to prevent or control the above-mentioned infections. The home-based care structure is generalised and integrated; it is not specific to a particular condition. Therefore, the VHWs have an oversight and support role to PCGs caring for different patients in the community including HBS patients.

Phase Two involved the development of educational interventions in the form of a job aid while Phase Three was the validation of the developed job aid by a panel of experts. This article reports the information on the last two phases of the study.

### Setting

The study was conducted in Mutasa district, Manicaland province, which is in the eastern region bordering Zimbabwe and Mozambique. Mutasa district covers almost 2774 square kilometres and is divided into 31 wards, which are further subdivided into villages. Each ward has at least 10 villages. Villages have households; Mutasa district has an estimated 42 479 households.^21^ The district according to Inpatient Morbidity and Mortality Information System (IMMIS), Manicaland province, had the highest incidence rate of stroke of 7.31 per 100 000 population when compared to the other six districts in the province in 2019.

### Intervention development

The authors adopted and adapted the systematic six steps in quality intervention development (6SQuID) by Wight et al.^22^ to guide the development of the educational interventions, which are:

1. Explaining in detail what the problem is and how it is caused: The cross-sectional study conducted among the PCGs and VHWs revealed the problem.
2. Detecting fundamental factors that can be adapted and who the beneficiaries are: It was noted that lack of knowledge among the PCGs emanated from VHWs not disseminating information to PCGs on stroke and its complications including infections; signs and symptoms of chest, skin and UTIs as well as measures to prevent and control these infections.
3. Determining interventions to effect change: Provision of basic educational material is likely to help improve PCGs’ practices in caring for HBS patients as well as their function and quality of life.
4. Describing how the intervention for change will be implemented: In view of the fact that technology may be costly and not readily available in a rural setting, it was imperative that the educational interventions are presented in the form of a job aid to guide training of PCGs of HBS patients on IPC.
5. Assessing effectiveness and adapting the intervention: In this study, the developed job aid was tested for validity, reliability, robustness and practicality by an eight-member panel of reviewers. Purposive sampling was utilised to select expert reviewers to validate the job aid. According to Creswell and Creswell,^23^ this sampling approach refers to purposeful identification of study participants because of their expertise in relation to the study. In Zimbabwe, there are very few individuals with expertise in infection, prevention and control; hence, the researcher approached those known to have the required expertise and were willing to participate in the validation process.

The following ensured elimination bias:

- Sampling was criterion based
- Use of a probability purposive sampling
- An independent coder was used to confirm data analysis
- Use of informed consent
- The expert panel was sent a brief context of the study in terms of the methodology used to gather and analyse information that leads to the development.

The reviewers with at least 5 years of experience in IPC-related work were purposively selected from senior IPC experts working in the public sector. The panel comprised two academics, one clinician in Public Health – Physician (a medical doctor), two registered community health nurses, and three IPC experts. According to Wight et al.,^22^ interventions are best developed through networking of relevant interdisciplinary teams and the affected population to ensure their effectiveness, relevancy, acceptability, practicality as well as evaluability. For the purpose of assessing the developed job aid, the panellists used a validating tool, which the researcher adapted from the Guideline for Reporting Evidence-based Practice Educational Interventions and Teaching (GREET) checklist (Online Appendix 1).^24^

6. Justification for a detailed evaluation: This stage was not done in this study because it was beyond the scope of the study.

The systematic 6SQuID educational model guides public health practitioners and researchers to develop effective interventions. With the assistance of two graphic designers for the development of the diagrams and annotations, the developed job aid was translated to a local language – Shona. The translation was done using forward and backward translation recommended in the WHO translation guidelines.^25,26^ The subtitles of the job aid contents were informed by the findings of the cross-sectional study conducted among PCGs and VHWs in Phase One of the main study.

### Validation process

Validation of the educational interventions was for the purpose to test robustness and practicality of the tool, thus a reviewer panel was established to test the tool for validity, reliability, relevancy, adequacy and effectiveness. The researcher used a validation tool in the form of a questionnaire that was adapted from the GREET checklist. The experts were individually contacted by telephone to introduce the request to them to be part of the reviewer panel that was followed by sending a formal request to each expert person in a form of an email of invitation that included a consent form for the purpose of an informed decision. Upon receipt of permission, the two documents, namely the job aid and the validating tool checklist (GREET), which included the 17 items cited in the Online Appendix 1 were e-mailed to each of the selected expert panel, for review.

The experts provided their opinions, which the researcher coded into numerical data for analysis by counting the number of votes or favours of each item of each domain. The validation tool had 32 items under five domains, and the responses were ranked using a five-point Likert scale from 1 to 5, with 1 representing very inadequate, 2 representing inadequate, 3 representing unsure, 4 representing adequate and 5 representing very adequate for the first four domains, and 1 representing strongly disagree, 2 representing disagree, 3 representing neutral, 4 representing agree and 5 representing strongly agree for the last domain.

### Ethical considerations

The principal investigator emphasised on voluntary participation, right to withdraw from participation, and that refusal would not lead to adverse consequences to the panel of experts. Confidentiality of data was strictly maintained throughout the study. An application for full ethical approval was made to the Nelson Mandela University Faculty Postgraduate Studies Committee (FPGSC) and the Medical Research Council of Zimbabwe and ethics consent was received on 10 April 2019 from the FPGSC, with ethics approval number H19-HEA-NUR-004, and ethics consent was received on 09 September 2019 from the Medical Research Council of Zimbabwe with ethics approval number MRCZ/A/2493.

### Data analysis

Validation scores and reviewers’ comments were analysed manually by both authors of this paper.

### Formulation of draft job aid

A combination of diagrams, pictures and texts were used to develop the job aid (Online Appendix 1). It was decided to develop a job aid comprising worksheets addressing the following:

- Scope of the job aid.
- Overview of stroke.
- Overview of IPC standard precautions.
- Common types of infections that occur most frequently in stroke patients, their causes, signs and symptoms.
- Prevention and control measures for chest infections, skin infections and UTIs in stroke patients.

In formulating the job aid, the authors took cognisance to ensure that the:

- content is relevant to the task at hand, thus prevention and control of chest, skin and UTIs in HBS patients.
- information is simple and concisely presented in short sentences to facilitate clarity and easy reference.
- job aid is written in the English language, which was backtranslated into Shona, a common and everyday language for the PCGs in Mutasa District; the language for instruction is Shona.
- drawings, diagrams or pictures are included in the worksheets to clarify information or provide more detail than words would allow. In this study, these visual elements will enhance learning and provide a quick reference to the PCGs by clarifying further information not understood.

### Comments and scoring from the expert panel reviewers

The comments and recommendations from the eight expert panel reviewers are presented according to the components used in the validation tool checklist. The scores as rated by the reviewers were calculated for each domain and are reflected in this section. The calculation for the domain score is explained in the first section, but thereafter only the final score is stated.

#### Background and participants’ information

The expert panel of reviewers agreed that the information on the title, target group and media of instruction of the jobaid was either adequately or very adequately explained. Based on the comments, the final job aid was accepted.

The values of scores given per item by the reviewers were used to calculate the domain score for this section and are shown in Table 1.

**TABLE 1:** Values of scores given by reviewers for background and participants’ information.

Reviewers	Item 1	Item 2	Item 3	Total
Reviewer 1	5	5	5	15
Reviewer 2	4	5	4	13
Reviewer 3	5	5	5	15
Reviewer 4	5	4	5	14
Reviewer 5	5	5	4	14
Reviewer 6	5	5	4	14
Reviewer 7	5	5	4	14
Reviewer 8	4	5	5	14
Total	38	39	36	113

*Source:* Adopted and adapted from the GREET checklist developed by Phillips et al.^24^

The values for the scores are:

Obtained score = 113

The minimum possible score for this section = 1 × 3 items × 8 reviewers = 24

The maximum possible score for this section = 5 × 3 items × 8 reviewers = 120

With the assistance of a statistician, the domain scores were calculated using the following mathematical formula:

$$\frac{\text{Obtained score}-\text{minimum possible score}}{\text{Maximum score}-\text{minimum possible score}}\times 100$$

The scaled domain for this section is:

113–24/120–24 × 100 = 89/96 × 100 = 92.7 = 93%

The higher the score rating that is obtained per domain, the greater the consensus, related to the domain assessed, thus validating the content of the job aid. The high scores indicate that the reviewers were positive about what the components of the job aid.

#### Educational process of the educational interventions

Assessment was done regarding the content of seven items namely: introduction, purpose of the educational interventions, definition of terms, references and layout – has a scaffolding approach, relevancy of the topics to the interventions and activities included in the interventions instrument (see Table 2).

**TABLE 2:** Comments on content of information by the reviewers.

Number	Item	Comments			
		Adequate	Very adequate	Unsure	Reviewers
1	Introduction	5	3	0	8
2	Purpose of the educational interventions	2	6	0	8
3	Definition of terms	3	4	1	8
4	References	1	7	0	8
5	Layout – has a scaffolding approach	5	3	0	8
6	Relevancy of the topics to the interventions	1	7	0	8
7	Activities included in the interventions instrument	3	4	1	8

*Source:* Adopted and adapted from the GREET checklist developed by Phillips et al.^24^

The content of the job aid was very adequate as reflected in Table 2, which shows that seven reviewers cited item 4 – references and item 6 – relevancy of the topics to interventions, six reviewers said item 2 – purpose of the educational interventions, four reviewers mentioned item 3 – definition of terms and item 7 – activities indicated in the interventions instrument. Five reviewers said content was adequate for item 1 – introduction and item 5 – layout – has a scaffolding approach. However, one of the reviewers recommended that item 7 in this section, namely, activities included in the intervention instrument, must be described more explicitly by indicating clearly what the PCGs are to do and not to do. Regarding item 3 – definition of terms, one reviewer commented unsure; the researcher sought clarification from the reviewer who pointed that the definition of stroke should not be the same as causes of stroke. The researcher corrected the definition. Based on the comments, the final job aid was adapted. The domain score for this section was 89%.

#### Overview information – 15 items for overview information on content assessed

Assessment was done of the content of 15 items, namely stroke, causes of infections in stroke patients, standard precautions on IPC, references to international and national IPC principles, standards and guidelines, causes of chest infections in HBS patients, practices to prevent and control chest infections in HBS patients, causes of skin infections in HBS patients, practices to prevent and control skin pressure ulcers in home-based patients, practices to prevent and control skin infections in home-based patients, causes of UTIs in HBS patients, practices to prevent and control UTIs in home-based patients, clarity, appropriateness, relevancy and simplicity of the contents. Seven members of the expert panel of reviewers accepted this section well in stating that the job aid was well designed, the language and content are clear, simple, relevant and appropriate for the target group. The instructions are easy to follow. However, two reviewers recommended regarding safe disposal of sharps to clearly state sharps’ container to be made of material, which does not allow needles to pierce through. Also, to indicate that the sharps container is to be disposed of when three quarters full. One reviewer recommended the use of a home-made container to be labelled ‘SHARPS’. In addition, one reviewer recommended that items addressing the definition of stroke, IPC standard precautions, causes of skin infections, practices to prevent and control chest, skin pressures and UTIs in HBS patients be stated more explicitly; contents to be made clearer and an image on paralysis of one side of body under signs and symptoms of stroke to be included. Furthermore, it was recommended to use clearer images on the job aid sections for patients propped in bed, having pain when urinating and having suprapubic pain. The final job aid was adapted as recommended by the reviewers. The domain score for this section was 86%.

#### Materials used in the educational interventions

The expert panel reviewers agreed that the information regarding the materials used in the educational interventions, which are worksheets translated into Shona, with diagrams and pictures added to enhance learning, for example, the item on the different types of protective attire such as gloves, their use and how to substitute them at home, is either adequately or very adequately explained. However, one reviewer recommended the inclusion of a more detailed description of alternative personal protective equipment (PPE) to use at home. The researcher felt mentioning use of plastics as substitute was adequate considering in a home setting options are usually limited. After a consensus discussion with the reviewer, it was decided not to include this aspect in the final job aid. The domain score for this section was 89%.

#### Effectiveness of educational interventions to fit with the target group’s perceived needs

Seven expert panel reviewers either agreed or strongly agreed that information in this section ensured understandability, acceptability, practicability and usability of the educational interventions by the PCGs; the educational interventions also ensured the capability to increase knowledge and practices of PCGs to prevent and control chest infections, skin infections and UTIs in HBS patients. Nonetheless, one reviewer was neutral in commenting effectiveness of the job aid to ensure Items 1 and 3, namely, understandability and practicality by the PCGs owing to the identified gaps mentioned above. The final job aid was adapted relying on the comments from the reviewers. The domain score for this section was 87%.

## Summary of the comments from the reviewers

Overall, the eight expert reviewers indicated that the job aid was simple, usable and relevant. The reviewers further commented that the tool was acceptable and understandable after editing information on measures to prevent and control chest and skin infections and UTIs as well as care of accessories, such as bedpans and tubes. The suggestions and recommendations by the expert panel reviewers were included in preparing the final job aid.

## Discussion

The limited knowledge and poor patient care practices towards prevention and controlling of complications associated with immobility of these patients were the results in phase one of the study. This finding was consistent with the research findings in the study of Mersal,^27^ which was about caregivers’ knowledge and practice regarding prevention of immobilisation complications in El-demerdash Hospital Cairo, Egypt. The educational job aid was then developed in order to bridge the identified concern about the PCGs in Zimbabwe. Similar to the situation of limited material for caregiver use in stroke patient care noted in Malaysia^28^ was also evident in Zimbabwe.

A job aid was recommended because the tool is developed with a specific task in mind, considering the procedure to be followed as well as tools necessary to complete the task.^29^ There is no definite format for the presentation of the job aid. Therefore, a job aid can be adopted and adapted to meet the needs of the learner in any setting. In the main study for this article, a combination of diagrams, pictures and texts is used to enhance understanding of the PCGs and improve their skills in caring for HBS patients.

According to Olmstead,^30^ examples of job aid format include step-by-step guides and process documentation (these instructional job aids guide employees through different steps required in completing a task); flowcharts (demonstrates visualisation of workflow) and checklists, which assist healthcare worker to check if they missed out any task. In the context of this study, the checklist was found to be appropriate, considering the literacy levels of primary caregivers in a rural set-up.

The discussion centres around the development, validation and benefits associated with the use of the job aid.

### Development process

Village health workers rated the information they gave to PCGs as inadequate or very inadequate for prevention of infections in HBS patients resulting in PCGs being ill prepared to care for them.^15^ Therefore, it is imperative that educational interventions in the form of a job aid were developed to equip PCGs with knowledge and skills necessary in caring for HBS patients.^31^ The 6SQuId model was appropriate to guide the development of the educational intervention because it provides more details and does not require technical skills and resources for intervention development, which makes the model more suitable to use as a guide than other existing models in the location of the study.^22^ Furthermore, the 6SQuID model guides public health practitioners and researchers to develop effective interventions as stroke is a public health problem. The application of the model ensures that the scarce public resources are optimally used.^22^ In the current study, the following six steps in quality educational intervention development were relevant and systematically guided the following development process:

**Step One** in the development of the intervention process defines the problem and its causes.^22^ Stroke is on the increase and the patients develop chest and skin infections as well as UTIs after discharge from hospital. Furthermore, there is no specific educational intervention on the IPC for PCGs caring for HBS patients to ensure standardisation in training in IPC. Absence of the necessary educational interventions results in vital information being omitted, leading to poorly executed care, which causes poor quality of life for stroke survivors. The PCGs of HBS patients in the study of this article lacked experience in caring for stroke patients and were not knowledgeable of stroke complications.^15^ A Malaysian study confirmed that informal stroke caregivers had limited knowledge of stroke complications^32^ and provision of education was necessary. A job aid is also designed for those with no experience; the job aid has simple instructions, diagrams and pictures, which make it easier for the PCGs of HBS patients to follow when providing nursing care.

**Step Two** identifies causal or contextual factors that are modifiable, those that have the greatest scope for change and who would benefit most.^22^ The VHWs did not give PCGs adequate information on stroke resulting in the PCGs not delivering quality care to HBS patients who then developed infections.^15,32^ In addition, a study conducted in Zimbabwe confirmed these findings that PCGs of HBS patients had either little or no healthcare training.^11^ Provision of job aid could help improve PCGs’ patient care practices as well as improve function and quality of life of the HBS patients. Mersal^27^ confirms that a job aid has the potential to improve the knowledge and skills of PCGs to prevent and control infections in HBS patients.

**Step Three** of the 6SQuID framework refers to mechanisms that will be used to effect change on the learners. Knowing the characteristics of both the learners and facilitators is essential to determine the appropriate teaching method.^22^ In the context of this study, face-to-face method will be used to educate the PCGs of HBS patients who might be either illiterate or semi illiterate. The main study findings of this article revealed that nearly two-thirds of the participating PCGs had been caring for HBS patients for less than 3 months. Spending limited time with the patient indicates that the majority of PCGs of HBS patients were new and inexperienced carers who were still learning about stroke and IPC measures involved. The findings of the same study showed that the PCGs of HBS patients had either little or no healthcare training, indicating the need for them to get information about IPC in stroke.^15^ Furthermore, low knowledge level of the PCGs of HBS patients may be attributed to lack of mentorship and preparation for the new stroke management role.^14^ Providing detailed, accurate and relevant IPC information through use of a job aid with worksheets could enhance PCGs’ understanding of stroke and measures to prevent and control chest and skin infections as well as UTIs. Therefore, job aids are necessary in training when an individual is either learning something for the first time or seeking to learn more.^30^ The training of PCGs is conducted in a systematic manner and the structure of job aid reduces the chances of making mistakes.^30^ Therefore, using a job aid will ensure consistency and standardisation in training of the PCGs of HBS patients.

**Step Four** describes the best mechanism to deliver the intervention.^22^ In this study, the educational interventions are presented in the form of a job aid, the reason being that technology may be expensive and not available in a rural setting to guide training of PCGs of HBS patients on IPC. Use of jobs aids in training helps the learners to retain information and are a reference tool for complex procedures. A study conducted in Nigeria in 2019 on effectiveness of job aids in training, confirmed that incorporating proven job aids into routine trainings is a low-cost strategy that can reinforce knowledge and help to retain information.^33^ Therefore, in the context of the main study, a job aid enables PCGs to acquire skills and knowledge during the training programme, which allows the PCGs to care competently and improve the HBS patients’ health outcomes.^31^

**Step Five** recommends that the developed intervention be tested for feasibility and effect possible adaptations.^22^ In this study, the developed job aid was tested for validity, reliability, robustness and practicality by an eight member panel of reviewers using a validating tool adapted from the GREET checklist.^24^ Comments from the reviewers were applied to refine the final job aid. The developed job aid is relevant, practical and would standardise and improve the quality training of PCGs to enable them to prevent and control chest, skin and UTIs among HBS patients. Kienapple^29^ cites that a job aid has clear, concise and easy to follow instructions that direct and guide implementers on what to do. Reference to a job aid improves the employee’s performance and enables an employee to complete a task accurately. The tool reduces avoidable mistakes by helping an employee remember what to do or be sure that all tasks have been completed as required.^29,30^

**Step Six** is the last step in the development of the intervention process strives to establish the evidence that the developed intervention is working as envisioned.^22^ The rigourous evaluation of the developed job aid with worksheets was not done in this study because it was beyond the scope of the study.

The study revealed that a job aid can address care challenges between health facility and community caregivers and can be used both during PCG training and patient care, which are key to the well-being of the HBS patients.

### Validation of educational intervention

In line with Wight et al.,^22^ it is critical that validity, reliability and robustness of the educational intervention are tested. In that regard, this study included the validation of the job aid. Dame^34^ posits that validation refers to the process of establishing reliability and validity of instruments as well as assessing whether they offer accurate and valuable information. The validation process forms the cornerstone for obtaining reliable information critical for decision-making and planning of patient care. In this study, a GREET checklist as described by Phillips^24^ was utilised to validate the job aid. The findings revealed that the job aid is simple and easily understood by PCGs. Kiennaple^29^ notes that a job aid should have clear, concise and easy to follow instructions that direct and guide implementers on what to do. Similar validation techniques have been utilised, for example, the Delphi survey involving international experts (Delphi panellists) who play a critical role in the evaluation of the job aid.^35^ The Delphi technique is a group process used to collect the opinions of experts on a particular subject.^36^ The technique is characterised by the geographical distance of the experts from one another, yet the validation process of this job aid only included local experts.

The developed job aid is the first validated tool on stroke care in Zimbabwe, and this resulted in lack of equivalent studies for comparison.

The study revealed that a job aid can address care challenges between health facility and community caregivers and can be used both during PCG training and patient care, which are key to the well-being of the HBS patients.

## Study limitations

The use of the purposive sampling method in the selection of the panel of experts to validate the job aid could result in low external validity of the findings; when recruiting the members of the panel, there might have been a selection bias. The selected sample of eight members could not be representative of the population of IPC practitioners, which may limit generalisation of the findings. However, the panel members were experts in the field of IPC. The study did not assess the usability and acceptability of the job aid among the caregivers because of limitations of financial resources.

## Conclusion

This study showed the development and validation of educational interventions to reduce infections among HBS patients in rural Zimbabwe. The developed job aid was validated with the participation of key stakeholders in IPC. Overall, the job aid addresses the knowledge barriers in IPC for PCGs and is important in closing the information gap. The validators of the developed educational interventions confirmed that the developed job aid is adequate for effective use by the lay home-based caregivers with low literacy rate. The validation of the job aid has demonstrated that its utilisation enhances efficiency in the delivery of home-based care services. Furthermore, the process established that the job aid is simple to use and understandable.
